# An unusual case of acute respiratory failure in a patient with pulmonary veins stenosis late after catheter ablation of atrial fibrillation: a case report and the review of the literature

**DOI:** 10.1186/s12890-015-0121-0

**Published:** 2015-10-24

**Authors:** Savino Spadaro, Sara Saturni, Delia Cadorin, Maria V. Colamussi, Matteo Bertini, Roberto Galeotti, Riccardo Cappato, Franco Ravenna, Carlo A. Volta

**Affiliations:** Department of Morphology, Surgery and Experimental Medicine, Section of Anesthesia and Intensive care, University of Ferrara, Via Aldo Moro, 8, Ferrara, 44121 Italy; Department of Morphology, Surgery and Experimental Medicine, Respiratory Medicine, S.Anna Hospital, Ferrara, Italy; Department of Cardiology, S. Anna Hospital, University of Ferrara, Ferrara, Italy; Department of Morphology, Surgery and Experimental Medicine, Vascular and interventional radiology Unit. S. Anna Hospital, Ferrara, Italy; Centro di Ricerca Aritmologia Clinica ed Elettrofisiologia, Milano, Rozzano Italy

**Keywords:** Acute respiratory failure, Haemoptysis, Pulmonary veins stenosis, Atrial fibrillation, Catheters ablation

## Abstract

**Background:**

Atrial fibrillation (AF) can be treated with percutaneous catheter ablation procedures into the left atrium. Pulmonary veins stenosis (PV) stenosis is a severe complication of this procedure.

**Case presentation:**

we report a case of late hemoptysis secondary to severe PV stenosis in a man who underwent AF ablation 9 months before onset of symptoms. He presented four episodes of bleeding and developed an acute respiratory failure (ARF). Parameters of respiratory mechanics and medical investigation did not show any abnormalities. Only computed tomography (CT) angiography showed stenosis of 3 out of 4 native PVs. PV balloon dilatation in all affected PVs and a stent was implanted in 1 of the 3 PVs with full restoration of respiratory function during 1 year follow-up.

**Conclusion:**

PV stenosis may be the underlying cause of recurrent haemoptysis after AF ablation in the presence of normal respiratory parameters. This diagnosis can be confirmed by means of CT angiography and magnetic resonance imaging can provide accurate localization of stenosis.

## Background

Massive haemoptysis is characterized by a relevant bronchial hemorrhage, usually quantified in 100–1000 ml in about 24 hours, and it is potentially life-threatening, representing a medical emergency [1]. When a patient presents haemoptysis the main clinical problems explored are infections, tumors, bronchiectasis, malformations, vasculitis, coagulative defects [1]. Haemoptysis as a complication due to cardiac ablation for atrial fibrillation (AF) is not even take into consideration [2]. However, AF is the most common clinically important cardiac arrhythmia occurring in 1–2 % of European population. Over 6 million Europeans suffer from this arrhythmia, which is increasing in frequency as the population ages [3, 4].

Usually, the first approach to AF is medical therapy or electric cardioversion. Catheter ablation strategies should be reserved for patients with AF, which remains symptomatic despite optimal medical therapy, or for paroxysmal AF in young patients with severe symptoms [4].

Percutaneous catheter ablation procedures involve the application of radiofrequency energy into the left atrium. Specifically, the technique consists of the electrical isolation of pulmonary veins (PV) from left atrium by encircling their ostium [3, 5]. Initially, almost 15 years ago, the electrical isolation of PV was performed applying radiofrequency energy, very close to the ostium, but 7–8 years ago the majority of centers began avoiding this technique and radiofrequency energy is now applied outside the ostium of PV thus reducing the risk of pulmonary vein stenosis (PVS) in long-term follow-up. Indeed, nowadays PVS after AF ablation is considered a rare complication. In general, the complications of this procedure performed with standard catheters include cardiac tamponade, systemic embolism, phrenic and vagus nerve lesion, atrial-esophageal fistula, pulmonary dysfunction and bleeding deriving from the anticoagulation required. PVS is a rare and potentially severe complication of this procedure [5] which occurs in 0.4 % to 1 % of patients, even in experienced centers [2, 3].

The clinical presentation, investigation, management, and outcome of this disease have not been completely explained.

This report describes a case of severe pulmonary vein stenosis associated with major haemoptysis and ARF requiring intensive treatment.

## Case presentation

A 40-year-old male, heavy smoker, occupational exposure to inhaling substances (his work consisted in floor tiling), was admitted to our intensive care unit on December 2013 because of major haemoptysis thus causing respiratory failure.

The patient’s medical history included hypertension and AF which began on December 2011, with no response to antiarrhythmic medications. In September 2012, the patient underwent catheter ablation for AF in another hospital. The procedure was conducted as conventionally with irrigated tip radiofrequency electrode. In particular, circumferential ablation about 1 cm away from PV orifice for all PVs was performed with maximum delivered power 35 watts at pre-set maximum temperature setting 38 °C. Since July 2013 he referred the onset of dyspnea and asthenia with several episodes of spontaneous haemoptysis. In October he was admitted to a medical ward. Complete blood count, coagulation study, arterial blood gases, bacterial and fungal cultures, virologic test, autoimmunity study, electrocardiogram, chest radiography and Positron Emission Tomography were carried out, but did not show any abnormalities. An echocardiogram performed on September 2013, showed a normal dimension and normal biventricular systolic function (estimated Ejection Fraction >70 %) and pulmonary artery pressure was estimated about 25–30 mmHg. Reumatological and Otolaryngological examinations were normal. He had no fever, chills, night sweats or weight loss. A thoraco-abdominal Computed Tomography study, performed in October 2013, documented the presence of mediastinal lymphoadenomegalia.

At the beginning of the flexible bronchial endoscopy, which was aimed at perform transbronchial fine needle aspiration of the node station 4R, 4 L and 7 (in order to exclude a proliferative pathology), the patient had hemorrhaged (about 400 ml in 30 minutes). The bleeding started immediately spreading from tracheobronchial mucosa. However, the bronchial tree anatomy was normal, and no evident hemorrhage source was found. Bleeding was copious and persistent, thus making the collection of a sample impossible and orotracheal intubation was required. Then, an angiography of the bronchial arteries was performed, but it did not reveal any active source of bleeding. However, a selective embolization was performed because of convoluted aspect of the bronchial arteries. Following the procedure, the patient was transferred to our intensive care unit in order to treat the respiratory failure.

**Fig. 1:**
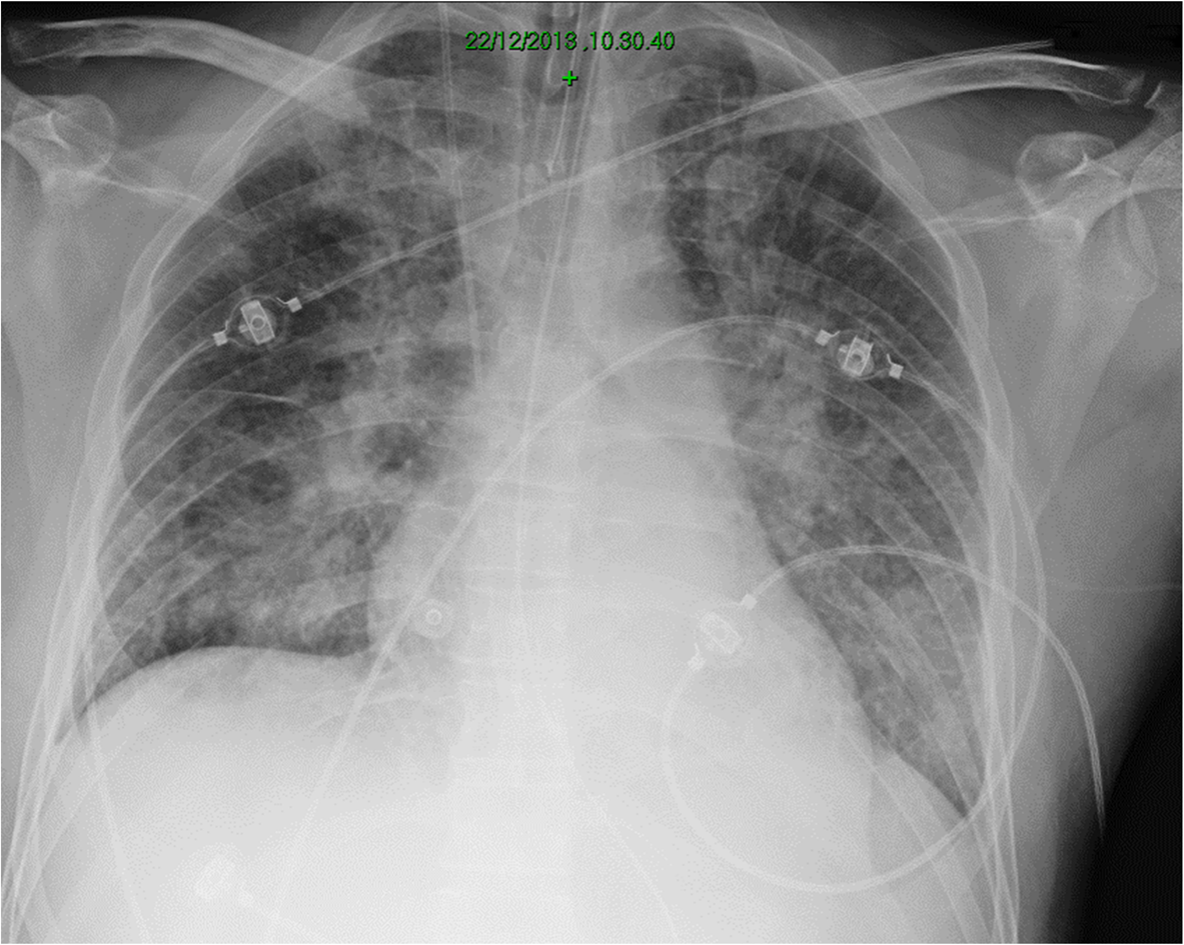
Chest X- ray made just before pronation (see text for further details)

Subsequently, he presented four more episodes of sudden major bleeding with acute desaturation. At the beginning, the oxygenation improved few hours after the bleeding events. Then the patient became severely hypoxic since the P_aO2_/F_IO2_ = 118 with a positive end expiratory pressure (PEEP) of 10 cmH_2_O. The pulmonary shunt fraction, calculated with the Automatic Lung Parameter Estimator system (ALPE Essential, Mermaid Care A/S, Nr. Sundby, Denmark) was about 16 %. A protective lung ventilation was established. The patient was paralyzed and ventilated in Volume controlled mode with a V_T_ of 5.7 ml/Kg and a PEEP of 10 cmH_2_O. Chest X-ray showing patchy bilateral infiltrates (Fig. 1). In order to improve oxygenation, the PEEP level was rise to 15 cmH_2_O and the shunt fraction decreased to 12 %. According to our protocol, we decided to prone him; the oxygenation further ameliorate since the shunt fraction decreased to 7 %. On the opposite, the static compliance of the respiratory system (Crs,st), equal to 52 ml/cmH_2_O, did not change by rising PEEP and by placing the patient in prone position. Cst, rs was calculated as V_T_/(plateau pressure – PEEPtot), where Plateau pressure and PEEPtot are the pressure registered after an end inspiratory/end expiratory occlusion of at least 5 sec, respectively. After 24 h of prone position, without further benefit in terms of oxygenation, the patient returned in supine position and the curarization was stopped. The shunt fraction was about 14 % with a PEEP of 10 cmH_2_O.

**Fig. 2:**
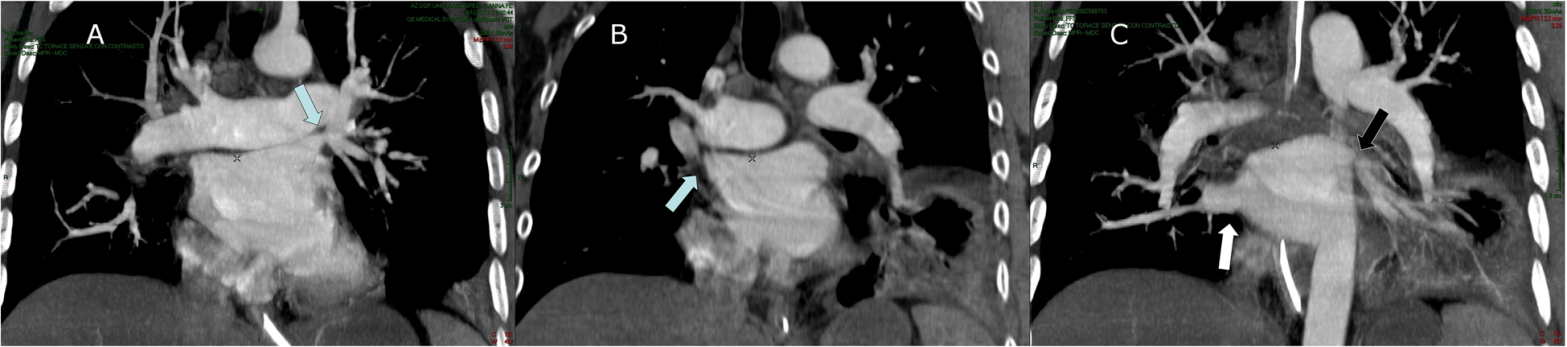
**a** Computed tomography angiography A-P projection showed the concentric severe stenosis of the left superior pulmonary vein (arrow). **b** CT angiography A-P projection showed 50 % stenosis of both right superior pulmonary (arrow). **c** CT angiography A-P projection showed ectatic right inferior vein (white arrow) and stenosis of left inferior pulmonary vein (black arrow)

The evident relationship between bleeding events and hypoxia imposed the re-analysis of the medical history of the patient. This helped us to notice the temporal correlation with the cardiac ablation. Indeed the latter could be responsible for severe complications, such as the PVS. Hence, to clarify this aspect, another Chest CT angiography was performed, at the seventh day of hospitalization. This showed the concentric severe stenosis of the right superior pulmonary vein (Fig. 2a) and the 50 % stenosis of both left pulmonary veins (Fig. 2b) while the right inferior vein appeared ectatic (Fig. 2c). The patient still intubated was then transferred to the Specialist Cardiologic Centre in order to perform balloon angioplasty with stent placement of the stenotic veins since the medical therapy was not able to reverse the ARF. One week after the procedure, the patient was successfully extubated.

## Discussion

PVS is a relatively rare condition, which can be both congenital or acquired. The congenital form is linked almost completely to congenital heart diseases and inadequate embryological cardiovascular connections, and evidences in childhood [6]. In adults, the pathology is even rarer, but approximately 15 years ago it appears in literature as a consequence of catheter ablation strategies for arrhythmias [6, 7], arising a bimodal age distribution [6].

The most common causes of acquired PVS in adult patients are radiofrequency ablation procedures for AF treatment. However, the underlying molecular mechanisms remain poorly defined, probably involving scarring, contraction of the venous wall and peri-adventitial inflammation or collagen deposition as a result of thermal injury, which may compromise or even occlude the lumen of PV [3, 5].

PVS is asymptomatic in most cases, especially when only one vein is involved. When clinically evident, the presentation varies depending several factors including: 1) the number of PV involved; 2) the severity of the lesions; 3) the response of the entire pulmonary vasculature to the lesion; 4) the time course of stenosis; 5) the clinical setting; and 6) the presence and extent of collateral vessels [3].

Patients with more extensive and severe involvement may present respiratory symptoms like dyspnea, orthopnea, cough, chest pain, recurrent pulmonary infections. Rarely, does an X-ray show bronchitis or pneumonia [3, 5].

Haemoptysis, the main symptom described in our case report, is relatively infrequent [3, 7]. Its etiopathogenesis has not yet been clarified, but the increase in venous pressure in the pre-stenotic zone could explain the lung tissue congestion and the risk of bleeding in this area [8].

Interestingly, our patient presented a severe hypoxemia requiring prone positioning [9]. This acute hypoxemic respiratory failure was interpreted as a consequence of repeated bleeding at the mucosal level capable of flooding the alveoli. It is true that the presence of blood in the lungs can be impetus for respiratory infections able to severely decrease oxygenation, such as pneumonia. However, we did not isolate bacteria in the bronchial aspirates or in the blood cultures; and the procalcitonin value was very low (0.04 ng/ml; n.v. <0.05). Further, although strictly speaking the acute respiratory failure of this patient could have been classifies as moderate ARDS, the lungs were not as stiff as in ARDS patients, since the values of static compliance were higher than those reported for patients with ARDS (33 ml/cmH_2_O) and even for patients ventilated for other diseases (44 ml/cmH_2_O) [10]. Hence, we thought that the principal diagnosis should have been different from the first that was hypothesized. Hemorrhagic pulmonary vasculitis was then considered in the differential diagnosis and a second CT angiography became mandatory.

The diagnosis of PVS may not be simple to identify, given the variability of clinical presentation and the atypical findings. Presence of dyspnea or cough after catheter ablation in the region of the PV should raise suspicions of PVS [3, 5]. Our case report suggests that even an ARF associated with nearly “normal” data of respiratory mechanics should imply a CT angiography and, to a lesser extent, Magnetic Resonance (MR) [11, 12] in order to exclude PVS. Indeed common radiological imaging (Chest X-ray or CT) is often irrelevant [7, 13]. Consequently, echocardiography should be routinely performed after AF ablation, because of the good feasibility, and the overall benefits in a population with increased risk of PVS development [6, 14]. Instead, CT angiography is a valid diagnostic tool, even if the resolution sometimes is not excellent, reducing the possibility to evaluate the stenosis degree [6, 15]. Furthermore, bronchoscopy has a major diagnostic role, especially when bleeding is the most relevant symptom, as underlined by international guidelines, which indicate the endoscopic evaluation as the most important instrument to investigate haemoptysis, after CT scans [16]. Nevertheless, there are cases in which bronchial endoscopy is not even taken into account, probably because of different clinical presentation (Table 1). However, the bronchoscopy value is particularly significant in differential diagnosis, in case of a normal CT, if the patient is high risk for lung carcinoma, or if symptoms continue [16].

**Table 1:** Cases of pulmonary vein stenosis with acute respiratory failure complicating trans catheter ablation of atrial fibrillation

Authors	N. of patients	Patients’symptoms	Diagnosis	Therapy	Similarities with our case	Differences with our case
Qureshi et al. 2003 [15]	19	● 17 cough	● CT scan	● One placement of an IVC filter	CT scan role	● Shorter time correlation with AF ablation
		● 12 haemoptysis				
		● 11 dyspnea on exertion				
		● 11 chest pain		● One patient with partial resection of the left lung		● Various different symptoms
		● 8 wheezing				
		● 7 dyspnea at rest				
		● 6 orthopnea				● No mention to bronchoscopy
		● 1 asymptomatic		● Angioplasty		
Saad et al. 2003 [20]	21	● 11 dyspnea	● CT scan	Angioplasty	● CT scan role	● Longer time correlation with AF ablation
		● 8 cough			● Treatment	
		● 8 asymptomatic				
		● 6 pleuritic chest pain				
		● 5 haemoptysis				● No mention to bronchoscopy
Packer et al. 2005 [13]	23	● 19 dyspnea on exertion	● Nuclear ventilation perfusion scan	Conservative treatment	● CT scan role	● Shorter time correlation with AF ablation
		● 10 cough	● CT imaging		● Treatment	
		● 7 dyspnea	● Angiography			● Different symptoms
		● 7 chest pain				
		● 3 asymptomatic				
		● 3 flu like symptoms				● No mention to bronchoscopy
		● 2 haemoptysis				
		● 2 decreased exercise tolerance				
		● 1 paroxysmal nocturnal dyspnea				
Calero Acuna et al. 2011 [7]	2	● Hemoptoic sputum and dyspnea on great exertion	Angiotomography of the pulmonary veins	● Angioplasty	● Haemoptysis	● Haemoptysis severity
				● Surgical intervention followed by stent placement		
		● Massive haemoptysis				
					● Unremarkable physical examinations and basic diagnostic exams	● No mention to bronchoscopy
						● More invasive treatment (surgical)
					● Diagnostic technique	
Mohsen et al. 2011 [19]	1	Mildly decreased exercise tolerance	CT angiography	Angioplasty and stenting	Treatment	● No haemoptysis
						● No mention to bronchoscopy
						● Shorter time correlation with AF ablation
Yun et al. 2012 [21]	1	● Haemoptysis,	● Magnetic Resonance angiography	Stent and anticoagulant therapy		● Shorter time correlation with AF ablation
		● Dyspnea on exertion				
		● Right chest pain				
			● CT scan			
						● No mention to bronchoscopy
Demelo Rodriguez et al. 2013 [8]	1	● Haemoptysis	● Angiography	Angioplasty	● Haemoptysis	● Longer time correlation with AF ablation
		● Dyspnoea on moderate exertion	● Bronchoscopy revealed a mucosa With petechiae which bled easily		● Orotracheal intubation and transfer to the intensive care unit	
					● Diagnostic technique and treatment	

PVS can be treated acutely with balloon dilatation of the PV, although the long-term outcome is uncertain [5]. Treatment options are currently limited, and re-stenosis after PV intervention has been described and considered relatively frequent [17]. Hence patients with PVS need careful follow-up because of the risk of recurrence, which occurs in 50 % of patients within 1 year [6].

Prevention of PVS is mainly related to placing the ablation site from inside to outside the orifice of the PV and reducing the ablation temperature and energy. This strategy should decrease the risk of PVS down to less than 1 % [18, 19].

## Conclusions

Clinicians should be aware of the possibility of PVS in patients who underwent ablation procedure for AF and those which present haemoptysis and acute hypoxemic respiratory failure. Hence pulmonary venous hypertension should be considered among the causes of ARF. Presence of dyspnea, cough, haemoptysis and acute respiratory failure after catheter ablation in the region of the PV should raise a high suspicion of PVS and a CT angiography should be performed since it can provide accurate location and evaluation of the extent of stenosis.

## Consent section

Written informed consent was obtained from the patient for publication of this case report and any accompanying images. A copy of the written consent is available for review by the Editor of this journal.

## Abbreviations

ARDS: Acute respiratory distress syndrome
ARF: Acute respiratory failure
AF: Atrial fibrillation
CT: Computed tomography
PEEP: Positive end expiratory pressure
PV: Pulmonary veins
PVS: Pulmonary veins stenosis
